# 2278. The Safety of Aztreonam vs. Ceftazidime in Patients with Penicillin Allergy: A Matched Cohort Study

**DOI:** 10.1093/ofid/ofad500.1900

**Published:** 2023-11-27

**Authors:** Jun Jie Tan, Peijun Yvonne Zhou, Nathalie Grace Sy Chua, Kai Chee Hung, Winnie Lee, Lai Wei Lee, Jia Le Lim, Yun Chun Shena Lim, Yixin Liew, Li Wen Loo, Narendran S/O Koomanan, Sarah Tang, Boon San Teoh, Daphne Yii, Siew Yee Thien, Pei Zhi Benjamin Cherng, Maciej Piotr Chlebicki, Andrea Kwa, Shimin Jasmine Chung

**Affiliations:** Singapore General Hospital, Singapore, Not Applicable, Singapore; Singapore General Hospital, Singapore, Not Applicable, Singapore; Singapore General Hospital, Singapore, Not Applicable, Singapore; Singapore General Hospital, Singapore, Not Applicable, Singapore; Singapore General Hospital, Singapore, Not Applicable, Singapore; Singapore General Hospital, Singapore, Not Applicable, Singapore; Singapore General Hospital, Singapore, Not Applicable, Singapore; Singapore General Hospital, Singapore, Not Applicable, Singapore; Singapore General Hospital, Singapore, Not Applicable, Singapore; Singapore General Hospital, Singapore, Not Applicable, Singapore; Singapore General Hospital, Singapore, Not Applicable, Singapore; Singapore General Hospital, Singapore, Not Applicable, Singapore; Singapore General Hospital, Singapore, Not Applicable, Singapore; Singapore General Hospital, Singapore, Not Applicable, Singapore; Singapore General Hospital, Singapore, Not Applicable, Singapore; Singapore General Hospital, Singapore, Not Applicable, Singapore; Singapore General Hospital, Singapore, Not Applicable, Singapore; Singapore General Hospital, Singapore, Not Applicable, Singapore; Singapore General Hospital, Singapore, Not Applicable, Singapore

## Abstract

**Background:**

Penicillin allergy (PA) is the most common drug allergy and is reported in up to 15% of hospitalized patients. Traditionally, aztreonam (AZT), a monobactam with low cross-reactivity to penicillin (PCN), has been recommended as an alternative antibiotic for patients with PA in our institutional empiric antibiotic guidelines. However, due to global AZT shortage in 2022, ceftazidime (CTZ), a cephalosporin with similar R1-side chain to AZT and low cross-reactivity to PCN, was used as a substitute for AZT. Fortuitously, this was cheaper - AZT (SGD $44.03 per 1g vial) vs CTZ (SGD $2.86 per 1g vial). There is a paucity of real-world data on the safety of CTZ in patients with PA. We proceeded to evaluate our experience with using CTZ as an alternative to AZT.

**Methods:**

This is a 1:1 matched, retrospective cohort study comparing patients with PA who received AZT from October 2022 to December 2022, with those who received CTZ from December 2022 to February 2023. They were matched based on severity of allergic reaction (AR) to PCN, age and gender. The risk of AR was stratified as low, medium or high based on the classification provided by the American Academy of Allergy, Asthma and Immunology. The severity of AR was based on the Delphi study grading system. The primary outcome was development of AR after initiation of AZT or CTZ. The secondary safety outcomes include hepatotoxicity and neurotoxicity.

**Figure:**
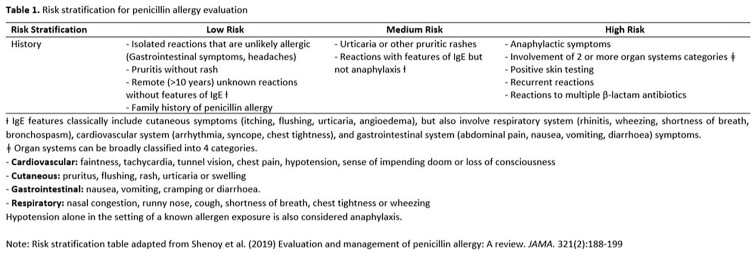

**Results:**

There were 132 patients: 52 patients (39.4%) were males, with a median age of 70 (IQR, 61 – 76) years, and majority were Chinese (n = 84 (63.6%)). The number of patients with low, medium and high risk of AR to PCN were 60 (45.4%), 54 (40.9%), and 18 (13.6%) respectively. The incidence of AR was similar in both groups: 1 patient (1.52%) in AZT arm vs 2 patients (3.03%) in CTZ arm (p = 1.0). The patient in the AZT arm had generalized urticaria (Grade 2). Both patients in the CTZ arm developed localized skin reaction (Grade 1). All 3 patients were deemed high risk of developing AR to PCN. Another patient in the AZT arm developed hepatotoxicity (raised alanine phosphatase and alanine transaminase), with a Naranjo score of 3 (possible adverse drug reaction).

**Figure:**
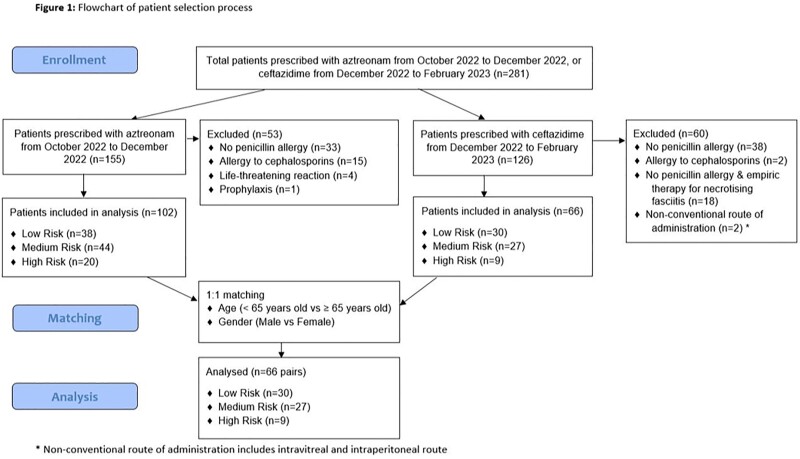

**Conclusion:**

CTZ is a safe alternative to AZT for patients labelled with PA, and may be a more cost-effective alternative in our setting.

**Disclosures:**

**All Authors**: No reported disclosures

